# Interplay between formation of photosynthetic complexes and expression of genes for iron–sulfur cluster assembly in *Rhodobacter sphaeroides*?

**DOI:** 10.1007/s11120-020-00789-w

**Published:** 2020-10-16

**Authors:** Xin Nie, Andreas Jäger, Janek Börner, Gabriele Klug

**Affiliations:** 1 Institute of Microbiology and Molecular Biology, University of Giessen, IFZ, Heinrich-Buff-Ring, 26-32 Germany; 2 Present Address: School of Basic Medical Sciences, Chengdu Medical College, Chengdu, 610500 China

**Keywords:** Bacterial photosynthesis, Iron–sulfur cluster assembly, *Isc-suf* operon, Gene regulation, Promoter activities

## Abstract

Formation of photosynthetic complexes leads to a higher demand for Fe–S clusters. We hypothesized that in the facultative phototrophic alpha-proteobacterium *Rhodobacter sphaeroides* expression of the *isc-suf* operon for Fe–S cluster formation may be increased under conditions that promote formation of photosynthetic complexes and that, vice versa, lack of the IscR regulator may also affect photosynthesis gene expression. To test this hypothesis, we monitored the activities of the *isc-suf* sense and anti-sense promoters under different growth conditions and in mutants which are impaired in formation of photosynthetic complexes. We also tested expression of photosynthesis genes in a mutant lacking the IscR regulator. Our results are not in agreement with a co-regulation of the Isc-Suf system and the photosynthetic apparatus at level of transcription. We provide evidence that, coordination of the systems occurs at post-transcriptional levels. Increased levels of *isc-suf* mRNAs under conditions promoting formation of photosynthetic complexes are due to higher RNA stability.

**Electronic supplementary material:**

The online version of this article (10.1007/s11120-020-00789-w) contains supplementary material, which is available to authorized users.

## Introduction

**Fig. 1:**
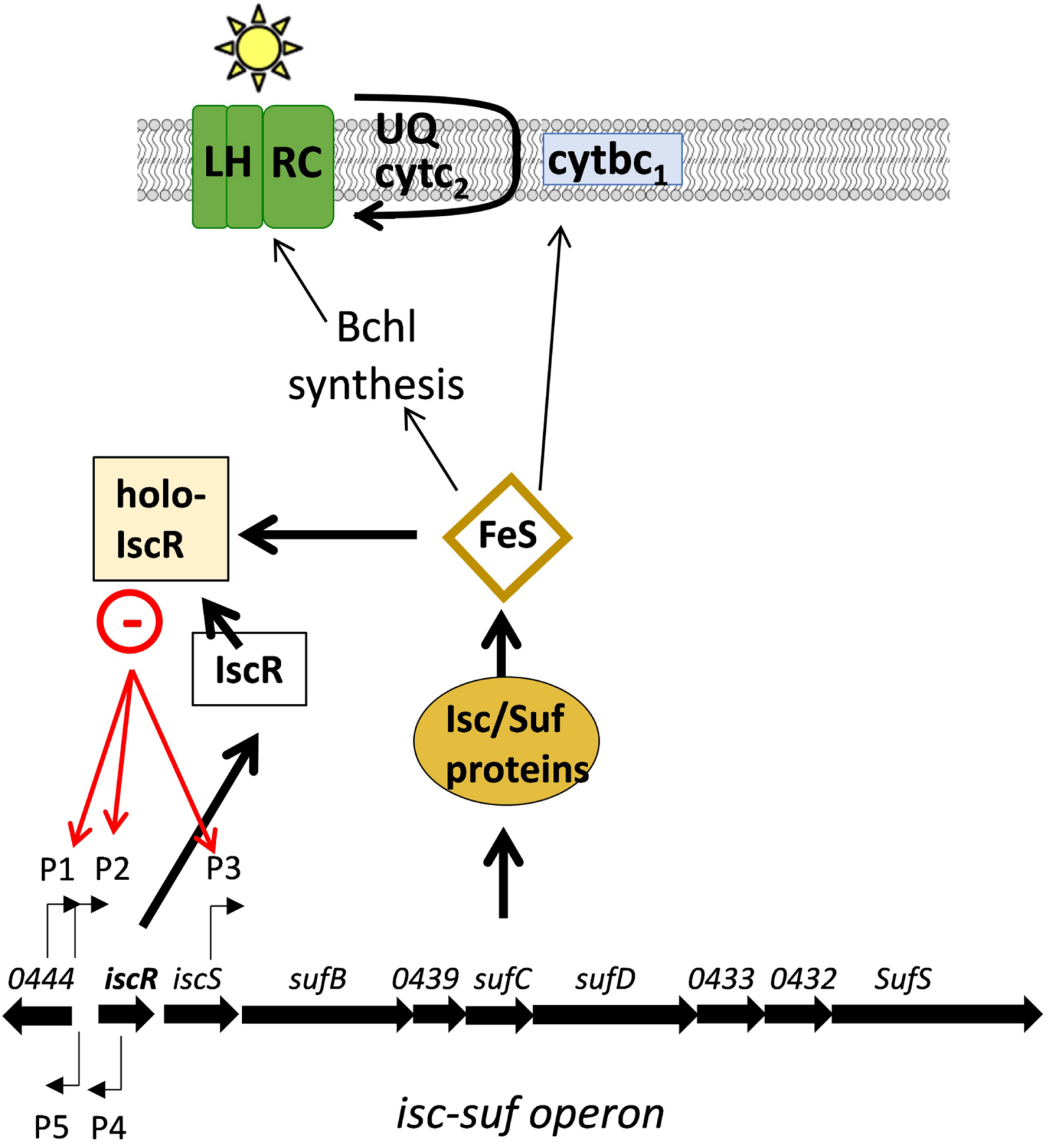
Fe–S clusters that are synthesized by the protein products of the *isc-suf* operon of *R. sphaeroides* are required for the formation of photosynthetic complexes and for photosynthetic electron transport. IscR is a main regulator of the *isc-suf* operon that can bind Fe–S (holo-IscR) and acts as a repressor

Iron–sulfur (Fe–S) clusters are required for manifold biological functions. They may function, e.g., in redox reactions, redox sensing, oxidative stress defense, DNA replication and repair, regulation of gene expression, or t-RNA modifications (Johnson et al. 2005; Py and Barras 2010). Fe–S clusters are also required for the process of anoxygenic photosynthesis as performed by *Rhodobacter* species. Magnesium chelatase and the dark-operative protochlorophyllide oxidoreductase that are involved in bacteriochlorophyll synthesis contain Fe–S clusters (Sirijovski et al. 2007; Selvi and Sharma 2008). Furthermore, the cytochrome *bc1* complex that is involved in chemotrophic and photosynthetic electron transport requires Fe–S clusters (Purvis et al. 1990; Trumpower 1990) (Fig. 1).

*Rhodobacter sphaeroides* is a facultative phototrophic bacterium that performs aerobic respiration if the oxygen level is high. Upon decrease of oxygen levels, it induces the formation of photosynthetic complexes that are assembled into intracytoplasmic membrane vesicles. The control of photosynthesis genes by oxygen has been intensively studied in the past. The two-component system PrrA/PrrB induces photosynthesis genes (for bacteriochlorophyll and carotenoid syntheses and for protein components of photosynthetic complexes) and the genes for the cytochrome *bc*_*1*_complex (*fbc* or *petABC* genes depending on annotation) upon decrease of oxygen tension (Imam et al. 2014). Photosynthesis genes are in addition controlled by the PpsR/AppA repressor / anti-repressor system that transmits redox- and light signals (Gomelsky and Kaplan 1997). At low oxygen tension AppA releases the repression by PpsR and photosynthesis gene expression is activated, while the interaction of AppA and PpsR at intermediate oxygen levels is influenced by light (Braatsch et al. 2002; Masuda and Bauer 2002; Han et al. 2007). As a consequence of this regulatory network, high amounts of photosynthetic complexes are formed upon drop of oxygen tension and assembled into newly formed membrane vesicles. The amount of RC and LHII proteins in the membranes increases by factors of 60 and more (Chory et al. 1984). Models of the chromophores position reaction center and light-harvesting complexes at the spherical part and the cytochrome *bc1* complexes at the base of the vesicles (Sener et al. 2007). One *bc*_*1*_ complex was suggested per two reaction centers (Crofts et al. 1983). When the proteomes of aerobically and phototrophically *Rhodobacter sphaeroides* 2.4.1 grown cultures were compared, a similar strong accumulation under phototrophic conditions was observed for reaction centers, *c*_*2*_ and *bc*_*1*_ type cytochromes (Callister et al. 2006). The levels of reaction centers and *bc*_*1*_ complexes in membrane vesicles are considered considerably higher than the levels of enzymes for carotenoid and bacteriochlorophyll synthesis (Zeng et al. 2007). The high amounts of new *bc1* complexes require high amounts of Fe–S clusters.

It was assumed that Fe–S assembly is an essential part of plastid functionality in plants based on the requirement for Fe–S proteins in multiple chloroplast processes (Kessler and Papenbrock 2005). In the cyanobacterium *Synechocystis sp. PCC 6803* SufR was identified as a negative regulator of the *sufBCDS* operon (Wang et al. 2004) that also contributes to regulation of reaction center biogenesis (Yu et al. 2003). A limited amount of the Suf system in *Synechocystis* by condition-controlled knock-down resulted in decreased chlorophyll contents and photosystem activities and an altered PSI/PSII ratio (Zang et al. 2017). The sRNA IsaR1 has an essential role in maintaining physiological levels of Fe–S cluster biogenesis proteins during iron deprivation in this bacterium (Georg et al. 2017). As a facultative phototrophic organism *R. sphaeroides* is an excellent model organism to analyze the effect of different levels of photosynthetic complexes on expression of genes for Fe–S assembly and vice versa.

*Rhodobacter sphaeroides* harbors a single operon for iron–sulfur cluster synthesis consisting of *iscR*, *iscS,* and *suf* genes (Fig. 1). The first gene of the operon encodes the IscR regulator that can coordinate an Fe–S cluster. The holoprotein (IscR with Fe–S) functions as a repressor of *isc* operon expression in *E. coli* (Giel et al. 2006) as well as *isc-suf* operon repressor in *R. sphaeroides* (Remes et al. 2015). We recently demonstrated that expression of the *isc-suf* genes is controlled by three sense (P1, P2, P3 in Fig. 1) and two anti-sense (P4, P5) promoters (Nie et al. 2019). P1 and P2 are located upstream of *iscR*, while P3 is located within the *iscS* gene and initiates transcripts spanning the *suf* genes. P4 generates transcripts anti-sense to the *iscR* mRNA. P5 initiates the RSP_0444 mRNA, which is partially anti-sense to the transcripts initiating at P1 and P2 (Fig. 1). A positive influence of both anti-sense promoters on expression of the *isc-suf* operon was observed (Nie et al. 2019). IscR binds to P1, P2 and P3 and is involved in iron-dependent and oxidative stress-dependent regulation of P2. The Irr protein, another known regulator of iron metabolism in *R. sphaeroides* (Peuser et al. 2012), also binds to the P3 promoter, while the redox regulator OxyR binds upstream of the anti-sense P5 promoter and affects its expression (Nie et al. 2019).

Based on the essential functions of and high demand for Fe–S clusters for photosynthesis it is likely that also facultative photosynthetic bacteria like *Rhodobacter* adjust Fe–S cluster production when photosynthetic complexes are formed; however, this issue has not yet been addressed. In this study, we analyzed whether altered amounts of photosynthetic complexes affect *isc-suf* expression and how lack of the IscR regulator would affect formation of photosynthetic complexes.

## Materials and methods

### Bacterial strains and growth conditions

Bacterial strains and plasmids are listed in Table S1. All *E. coli* strains were cultivated in Standard I medium at 37 °C, either in liquid culture by shaking at 180 rpm or on solid growth medium, which contained 1.6% (w/v) agar. Depending on the cultivated strain the antibiotic tetracycline (20 μg / ml) was added to the solid and liquid growth media. *R. sphaeroides* strains were cultivated in 50 ml Erlenmeyer flasks containing 40 ml minimal malate medium (Remes et al. 2014) with continuous shaking at 32 °C (low oxygen with dissolved oxygen concentration of 25–30 μM). High oxygen conditions with 160 to 180 μM dissolved oxygen were achieved by incubating 25 ml of culture in 100 ml Erlenmeyer baffled flasks. For phototrophic growth Meplat bottles were filled with culture to the top, sealed and illuminated (60 W/m^2^ of light). For anaerobic growth in the dark, DMSO (60 mM) was provided as terminal electron acceptor to cultures in Meplat bottles. Cells were harvested at an OD_660_ of 0.5–0.6. Antibiotics were added to the liquid and solid growth media depending on the cultivated strain at the following concentrations: kanamycin (25 μg ml^−1^), trimethoprim (50 μg ml^−1^), tetracycline (2 μg ml^−1^).

### Constructions of promoter fusion plasmids

Fragments with lengths ranging from 120 to 1735 bp containing one of the putative five different single promoters or combined promoters of the *isc-suf* operon, respectively, were amplified by PCR with primers listed in Table S2. The PCR product was ligated into pJET1.2/blunt cloning vector (Qiagen) and then transferred into *E. coli* JM109. After confirming the correct sequence, the promoter fragment cut from the sequenced cloning vector by XbaI or PstI and subsequently ligated into the transcriptional *lacZ* fusion vector pBBR1-MCS3-LacZ (Kovach et al. 1995) as described in (Nie et al. 2019).

### ß-Galactosidase-measurements

ß-Galactosidase activity of transcriptional fusions was measured by hydrolysis of O-nitrophenyl-ß-d-galactopyranoside (ONPG) and expressed as Miller Units as described in (Nie et al. 2019).

### RNA isolation and quantification

20 ml of *R. sphaero*ides cells were harvested by centrifugation when an OD_660_ of 0.5 was reached. Total RNA for quantitative RT-PCR was isolated by using the peqGOLDTriFast kit (Peqlab) as described by the manufacturer. Remaining traces of DNA were removed by TURBO DNaseI (Invitrogen). PCR targeting *gloB* (RSP_0799) with the primers listed in Table S2 was performed to confirm the absence of DNA. Quantitative RT-PCR was performed in a Bio-Rad CFX96 Real-Time system as described in our previous study (Remes et al. 2014). The reference gene *rpoZ* encoding the ω-subunit of RNA polymerase of *R. sphaeroides* was used to normalize the mRNA expression levels (Zeller and Klug 2004) according to the formula given by Pfaffl (Pfaffl 2001). Primers are listed in Table S2.

Library preparation and sequencing, read mapping and quantification by DEseq for RNAseq is described in (Remes et al. 2014), GEO accession number for RNAseq data: GSE47182.

### Whole cell spectra

10 ml of liquid cultures in the exponential growth phase (OD_660nm_ about 0.5) were concentrated to 1 ml and spectra were monitored on a Specord 50 plus photometer (Analytik Jena) by placing the cuvettes in the position close to the photomultiplier and using 1 ml of RÄ-medium as reference.

### Quantification of photopigments

Cells from 1 ml of culture in the exponential growth phase (OD_660nm_ is about 0.5) were harvested by centrifugation (13,000 rpm, 5 min, RT) and the pellets were resuspended in 50 μl H_2_O. The photopigments were extracted with 500 μl of a mixture of acetone and methanol (7:2, v/v) from the pellets. After vortexing the samples for 10 s and centrifugation (13,000 rpm, 5 min, RT), the absorption of the supernatant is measured at 484 nm and 770 nm. The mixture of acetone and methanol (7:2) is used as reference. The contents of bacteriochlorophyll and carotenoids, respectively, are calculated using the extinction coefficients of 76 mM^−1^ cm^−1^ and 128 mM^−1^ cm^−1^ at 770 nm and 484 nm (Clayton 1966).

### Half-life measurement

The *R. sphaeroides* cultures were incubated under microaerobic and anaerobic conditions. Samples for RNA isolation were taken directly before addition of rifampicin (final concentration is 0.2 mg/ml) and every 5 min until 60 min after the addition. qRT-PCR was used for RNA quantification to calculate the half-life of *iscR* mRNA.

## Results

### Influence of growth conditions that induce formation of photosynthetic complexes on expression of *isc-suf* genes

**Fig. 2:**
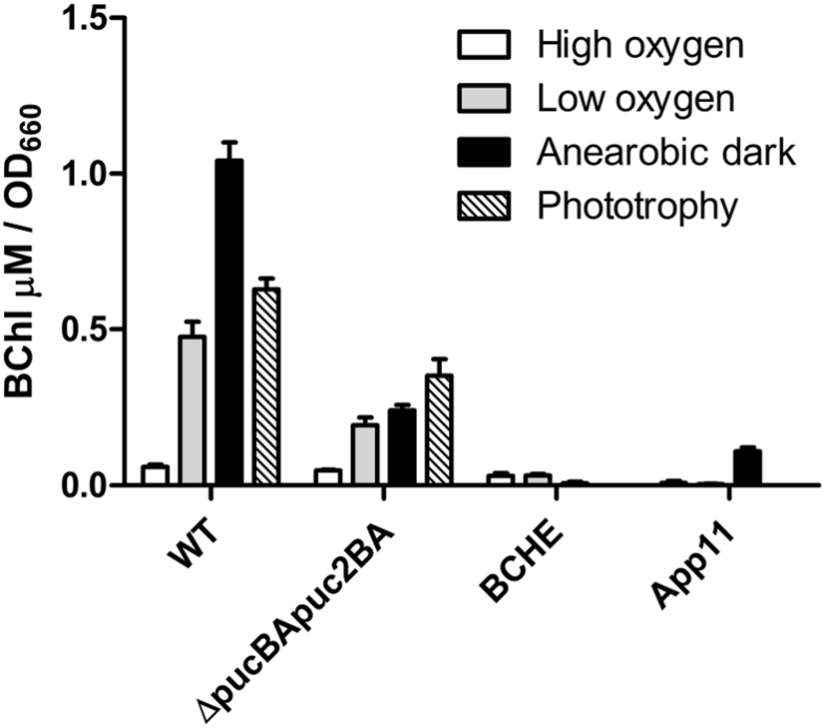
Content of bacteriochlorophyll (normalized to the optical density) in different strains grown under different conditions. The bars represent the average of technical duplicates from biological triplicates, the error bars represent the standard deviation

Environmental conditions, especially the oxygen tension, have a strong impact on the amounts of photosynthetic complexes formed by *R. sphaeroides*. We chose the following conditions for growth and further analysis: i) high oxygen level (aerobic growth, 160 to 180 μM dissolved oxygen), ii) low oxygen levels (microaerobic growth, 25–30 μM dissolved oxygen), iii) anaerobic growth in the dark with DMSO as terminal electron acceptor for anaerobic respiration, iv) anaerobic growth in the light (phototrophic growth 60 W/m^2^of light). Only illumination allows ATP production by photosynthesis (phototrophic growth). Fig. S1 shows representative spectra for wild type cultures grown under these conditions to the identical OD_660_ of about 0.5–0.6 (anaerobic dark 0.2–0.25, spectra normalized to OD). Since all bacteriochlorophyll in the cell is bound to photosynthetic complexes, its levels represent the different amounts of photosynthetic complexes (Fig. 2). Considerably higher levels of photosynthetic complexes are formed under anaerobic dark conditions (about 17.4-fold more bacteriochlorophyll) than under aerobic conditions or under low oxygen conditions (about 2.2-fold more bacteriochlorophyll). Oxidative stress leads to the destruction of Fe–S clusters and activation of genes for Fe–S cluster assembly in *E. coli* (Lee et al. 2008). Transition from phototrophic to aerobic conditions resulted in transient increase of *isc-suf* expression in *R. sphaeroides* (Arai et al. 2008). Therefore, it is important to compare growth conditions leading to different amounts of photosynthetic complexes without oxidative stress. For this reason, stronger aeration of the cultures was avoided and also microaerobic conditions were included in this study. Aerobic respiration is used under aerobic and microaerobic conditions for energy production, so Fe–S containing enzymes for this process are required under both conditions.

**Fig. 3:**
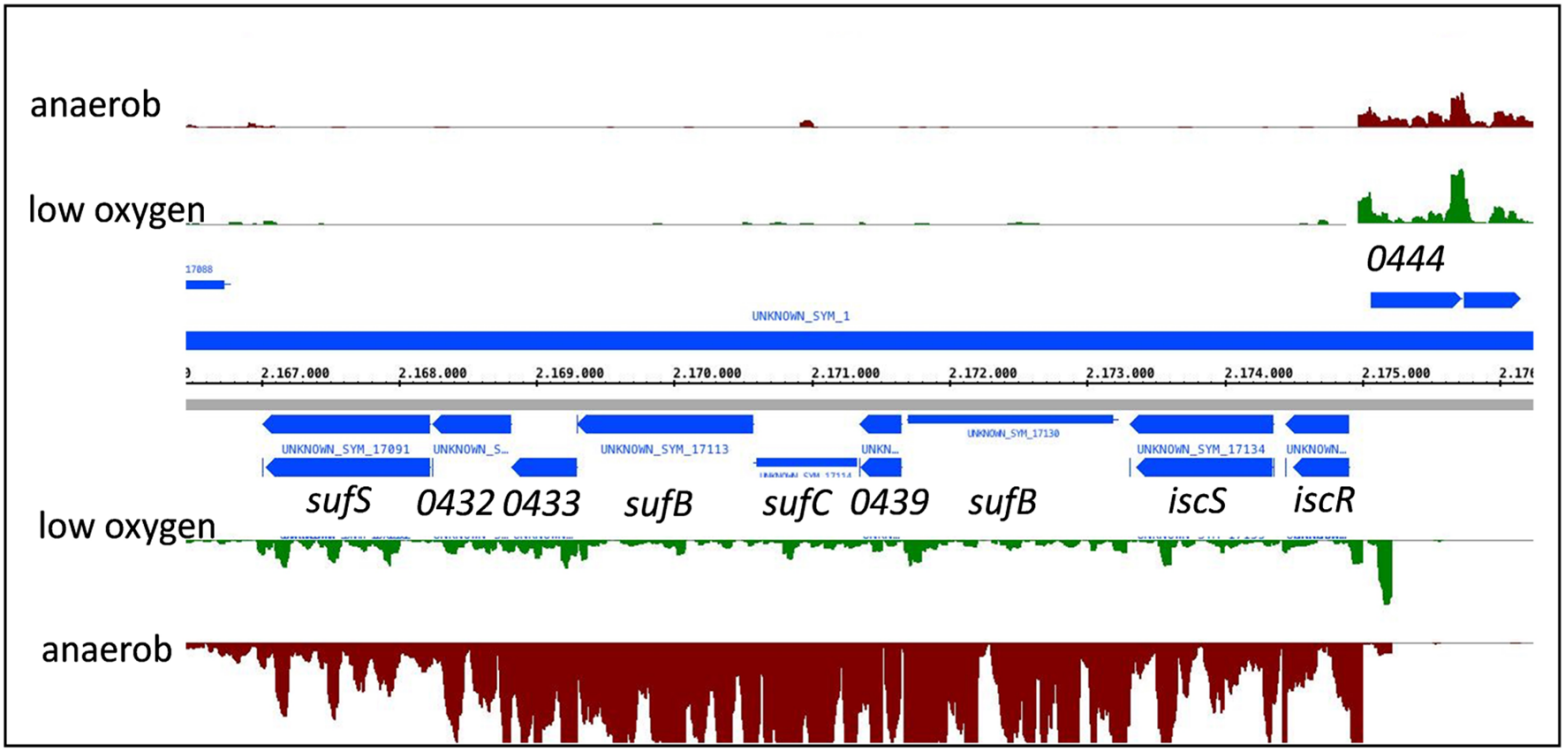
Screen shot from the Integrated Genome Browser visualizing the normalized read numbers as determined by RNAseq (Remes et al. 2015). The same scale was chosen for all conditions. The *isc-suf* genes are located on the minus strand. RSP_0444 on the plus strand is not related to iron–sulfur cluster assembly

In a previous study we used RNAseq to compare RNA levels in *R. sphaeroides* wild type 2.4.1 grown under microaerobic conditions (25–30 µM oxygen) or anaerobically in the dark with DMSO as terminal electron acceptor (Remes et al. 2014). Figure 3 shows a screen shot from the Integrated Genome Browser for the *isc-suf* operon. As reported in (Remes et al. 2014), in presence of iron *isc-suf* mRNAs are much more abundant during anaerobic growth, when high amounts of photosynthetic complexes are formed (Fig. S1). Quantification of the normalized read numbers by the DEseq tool revealed 2.3-fold (*sufS*) to 7.4-fold (*sufB, sufC, sufD*) higher levels under anaerobic conditions. Figure 3 also shows that neither oxygen nor DMSO, the electron acceptor under anaerobic conditions, influence the levels of mRNA transcribed from RSP_0444 (not related to Fe–S assembly). The unchanged read numbers of RSP_0444 also demonstrate that the same amounts of RNA were present in both samples.

**Fig. 4:**
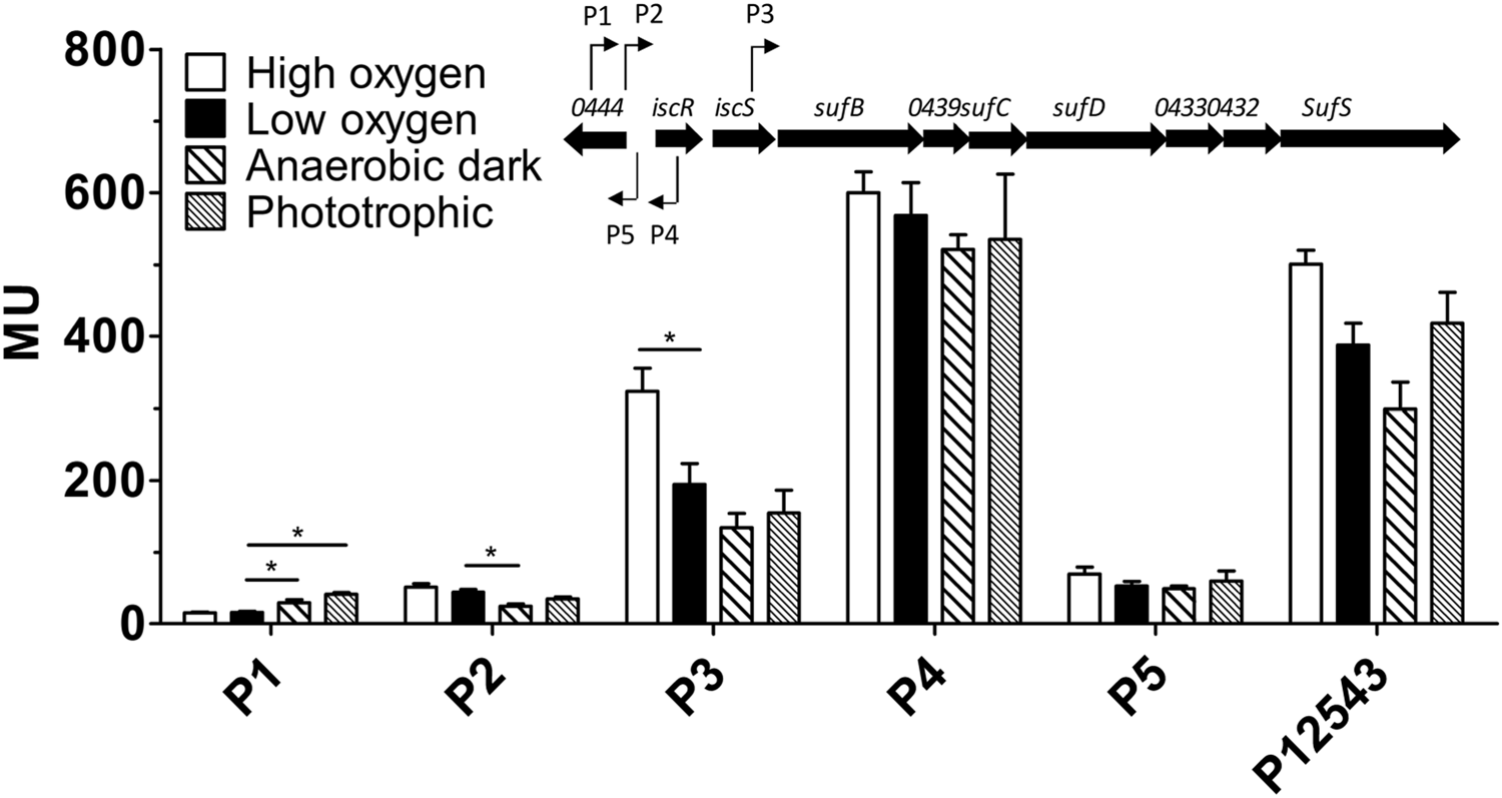
Influence of growth conditions that affect formation of photosynthetic complexes on activity of the individual *isc-suf* promoters and on a fusion of P3 to *lacZ* that contains all other promoters upstream of P3 (P12543). Details on the *lacZ* fusion constructs are provided in Fig. S2. The bars present the average of technical duplicates from biological triplicates, the error bars represent the standard deviation. *The difference between the values for different conditions is > 1.5-fold with a p-value of < 0.01

We tested the activity of the *isc-suf* promoters in exponential phase cells from wild type cultures grown under the different conditions. Fig. S2 provides details on the reporter fusions used in this study. Significantly increased activity under anaerobic dark conditions was only observed for P1 (1.8-fold compared to low oxygen tension), which is the weakest of the five promoters (Fig. 4). P2 activity was significantly decreased under anaerobic dark conditions (1.8-fold compared to low oxygen). For all other promoters including the fusion to P3 with a long upstream fragment and all promoters present (P12543), no significant change (p ≥ 0.01) or slight decrease (< 1.5-fold) was determined under anaerobic conditions compared to incubation under low oxygen conditions. In agreement with previous data (Nie et al. 2019), P3 showed increased activity (1.7-fold compared to low oxygen) under high oxygen tension compared to low oxygen.

These data reveal that the formation of high amounts of photosynthetic complexes under anaerobic conditions does not lead to transcriptional activation of *isc-suf* genes. For all promoters the activity values for anaerobic dark conditions were similar to phototrophic conditions. Since some of the mutants investigated in this study are unable to grow phototrophically, we applied the anaerobic dark incubation for conditions resulting in high amounts of photosynthetic complexes.

**Fig. 5:**
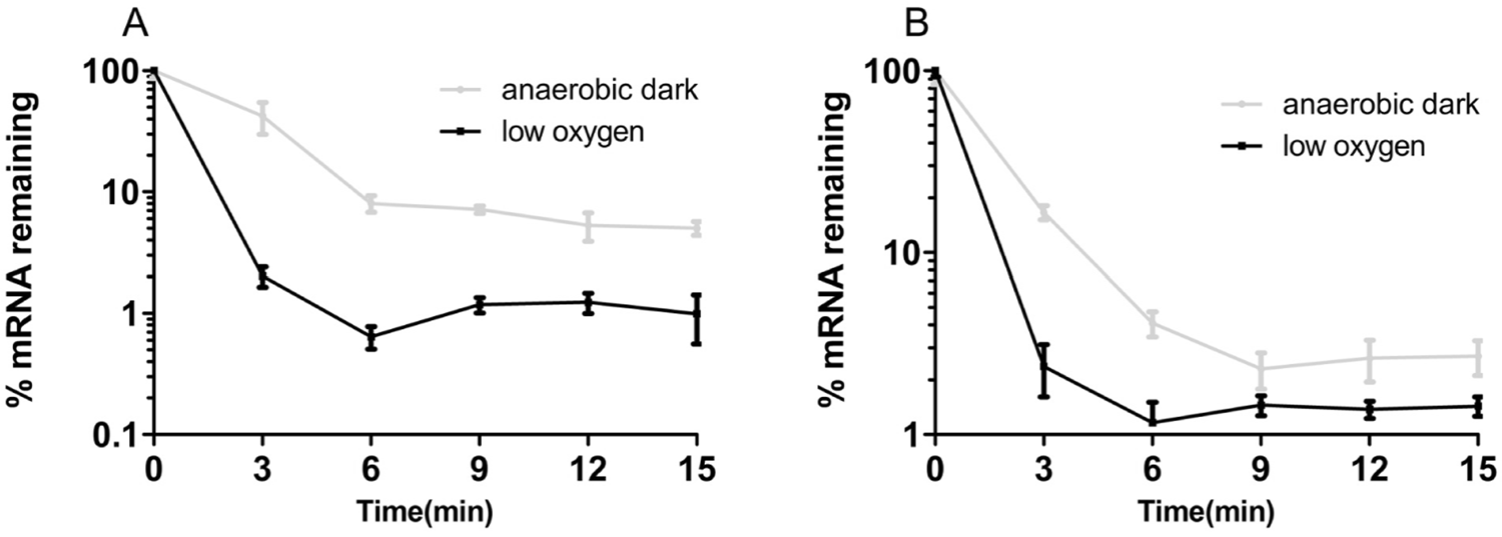
Stability of the *iscR* and *sufB* mRNA. The RNA levels were quantified by real-time RT-PCR at different time points after addition of rifampicin. The average of three measurements from biological triplicates is shown and the standard deviation is indicated. A: *iscR*. B: *sufB*

Since the RNAseq data clearly revealed increased *isc-suf* mRNA levels in absence of oxygen, but *isc-suf* promoter activities did not, we assumed that the different mRNA levels are due to altered stability of the mRNA. To test this assumption, we isolated total RNA at different time points after addition of rifampicin which stops initiation of transcription in bacteria. The RNA levels were quantified by real-time RT-PCR. Indeed, increased half-lives for the *iscR* and *sufB* mRNA segments were confirmed from about 1 min (about 0.8–1.0 min) under microaerobic conditions to about 2 min (1.8–2.1 min) under anaerobic dark conditions (Fig. 5).

### Activity of *isc-suf* promoters in mutants affected in synthesis of photosynthetic complexes

Under the different growth conditions used for our analyses not only the amounts of photosynthetic complexes change but also other factors in the cellular environment like redox state of proteins or activity of oxygen and light-dependent regulators. Therefore, we decided to also compare activity of the *isc-suf* promoters in mutants with altered levels or composition of photosynthetic complexes (Table S1) to that of the isogenic wild type under identical growth conditions. Fig. S3 shows spectra and Fig. 2 bacteriochlorophyll levels for the different mutant strains used in this study. All bacteriochlorophyll is bound to pigment-binding proteins. Consequently, the bacteriochlorophyll levels reflect the amounts of photosynthetic complexes.

The majority of bacteriochlorophyll is bound to the LHII complex (absorbance at 800 and 850 nm). Mutant 2.4.1Δ*pucBApuc2BA* has both operons for the proteins of the LHII complex deleted from the chromosome and thus forms only reaction center (absorbance at 803 nm) and LHI complex (absorbance at 870 nm). As a consequence, mutant 2.4.1Δ*pucBApuc2BA* contains 4.3-fold less bacteriochlorophyll compared to the wild type under anaerobic dark conditions (Fig. 2). Mutant BCHE has the *bchE* gene for Mg-protoporphyrin IX monomethyl ester oxidative cyclase deleted and only minor amounts of photosynthetic complexes are formed (more than 100-fold less bacteriochlorophyll than the wild type under anaerobic dark conditions, Fig. 2). Strain App11 has the *appA* gene deleted. Since the anti-repressor AppA is not present, the repressor PpsR is strongly reducing the expression of photosynthesis genes and consequently the amounts of photosynthetic complexes (Gomelsky and Kaplan 1997). Strain App11 contains about 9.5-fold less bacteriochlorophyll than the wild type under anaerobic dark conditions (Fig. 2). Since strains BCHE, and App11 cannot grow photosynthetically, phototrophic growth conditions could not be used for comparing promoter activities between mutant strains and wild type.

**Fig. 6:**
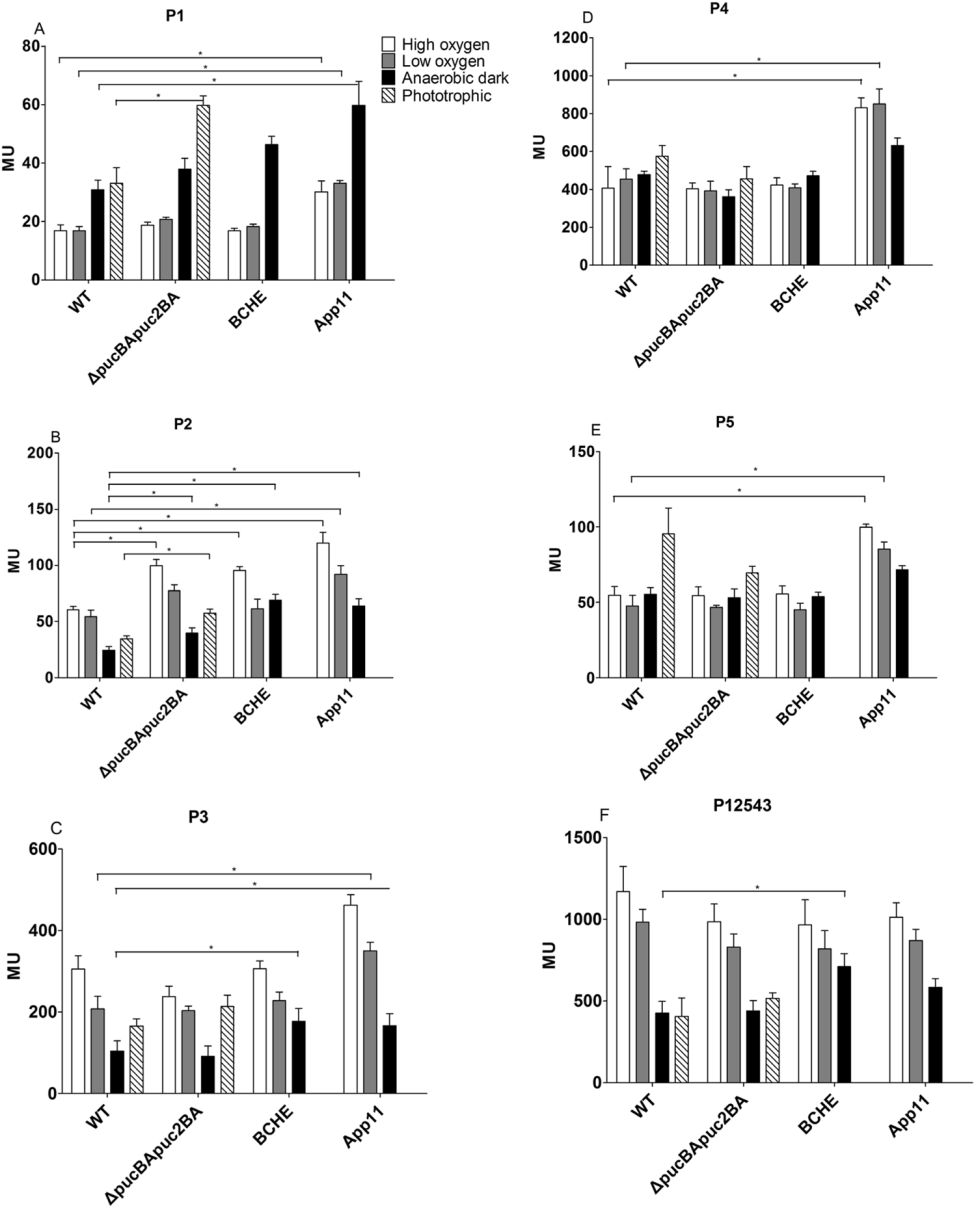
Activity of the individual promoters and of P12543 as determined by *lacZ* reporter assays and quantified by measuring the ß-galactosidase activity in Miller Units (MU) in the wild type and mutant strains under different growth conditions. The bars represent the average of technical duplicates from biological triplicates, the standard deviation is indicated. *The difference between the values for different conditions is > 1.5-fold with a p-value of < 0.01

Activities of P1 in strains 2.4.1Δ*pucBApuc2BA* and BCHE under aerobic or microaerobic conditions were similar to those in the wild type (Fig. 6a), while it was slightly increased (maximal 1.5-fold) in both mutants under anaerobic conditions. In the mutant lacking AppA (App11) P1 activity was increased (1.8 to 2.0-fold) under all conditions. P2 is the main promoter for *iscRS* transcription (Fig. 1). For P2 we found increased activity under high oxygen for all mutants (1.6-fold for Δ*pucBApuc2BA*, 1.6-fold for BCHE, 2.0-fold for App11). Strongest increase in P2 activity (1.7 to 2.6-fold) under all conditions was observed for the strain lacking AppA (Fig. 6b). P3, P4, and P5 showed only slight variations in activity between wild type and strains 2.4.1Δ*pucBApuc2BA* and BCHE (Fig. 6c–e). There were, however, marked difference for the mutants lacking AppA: activity of P3 that initiates *suf* gene transcription was increased in App11 under aerobic and microaerobic conditions. Activity of anti-sense promoters P4 and P5 was also increased in strain App11 under these conditions (1.8 to 2.0-fold). We also monitored activity of a P3 promoter fusion that had all other upstream promoters (P12543) present (Fig. 6f). In strains lacking bacteriochlorophyll, P12543 showed slightly higher activity (1.7-fold) under anaerobic dark conditions than the wild type.

In summary, we detected some differences in activity of the *isc-suf* promoters in the tested mutants, but higher amounts of photosynthetic complexes did not correlate with higher activity of *isc-suf* promoters. So, any co-regulation between formation of iron–sulfur clusters and formation of photosynthetic complexes would need to occur at post-transcriptional level. This may also include altered *isc-suf* mRNA stabilities as shown in Fig. 5 for anaerobic dark conditions.

### Effect of an *iscR* deletion on levels of photosynthetic mRNAs

**Fig. 7:**
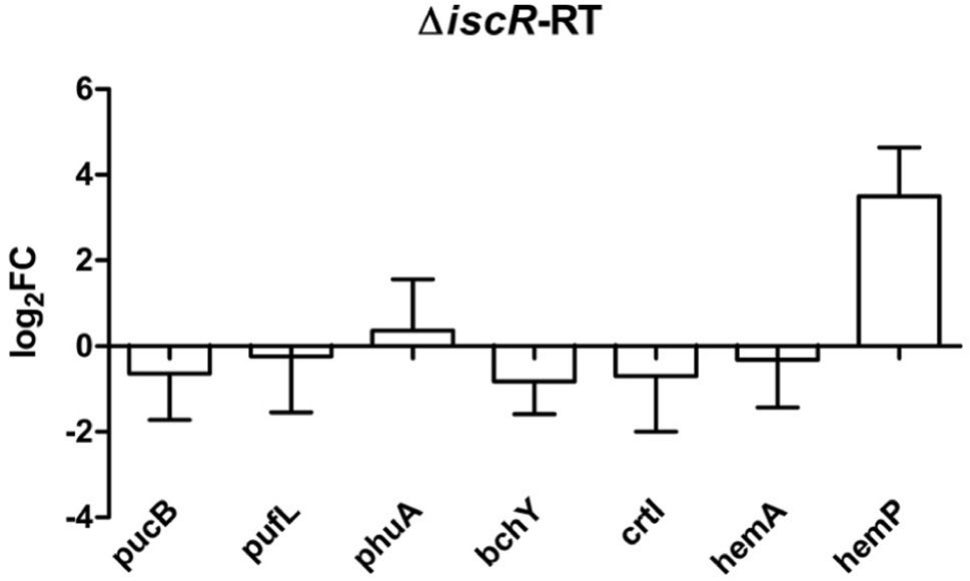
Levels of mRNAs for selected photosynthesis genes as determined by real-time RT-PCR in an *R. sphaeroides* strain lacking *iscR* compared to the wild type under microaerobic conditions. The bars represent the average of technical duplicates from biological triplicates and error bars depict the standard deviation

IscR is a regulator of the *isc-suf* operon but also of several other genes involved in iron metabolism. A transcriptome study revealed that a lack of IscR (deletion of part of the coding sequence and insertion of an antibiotic cassette without transcriptional terminators) leads to slightly decreased *suf* mRNA levels (about 1.5-fold) and had no effect on photosynthesis gene expression (Remes et al. 2015). IscR is a repressor that affects P2 and P3 activity (Nie et al. 2019). Due to the deletion of *iscR*, *iscS* mRNA levels were also decreased and the positive effect of transcription initiating at P1 and P2 on expression of *suf* genes was lost. When we followed formation of the photosynthetic apparatus in this mutant, we observed reduced amounts of pigment-protein complexes compared to the wild type (Fig. S4). We quantified the levels of photosynthesis mRNAs in the *iscR* mutant by real-time RT-PCR (Fig. 7), which is more sensitive and has a higher dynamic range than the microarrays. In agreement with the previous microarray results, we did not detect significant differences in expression levels between the mutant and the wild type. As a control we also quantified the *hemP* (RSP_6006) mRNA level in both strains and found much higher amounts in the mutant, as observed in the previous microarray study. IscR was shown to bind to the *hemP* promoter region (Remes et al. 2015). While HemA catalyzes the synthesis of aminolevulinic acid, a precursor of protoporphyrin, HemP is a small hemin uptake protein that is not directly related to photosynthesis. We conclude that deletion of IscR does not lead to decreased amounts of photosynthetic complexes by affecting the levels of photosynthetic mRNAs. It is conceivable that protein synthesis or stability are affected, which not sufficient bacteriochlorophyll is present or that formation of the cytochrome *bc1* complex is impeded as a consequence of the IscR effects on Fe–S assembly.

## Discussion

The higher demand for Fe–S clusters under conditions that favor formation of photosynthetic complexes in *R. sphaeroides* raised the question whether the amounts of photosynthetic complexes affect *isc-suf* operon expression and/or vice versa. Since the IscR regulator is a Fe–S sensor, it is conceivable that the high demand of photosynthetic complexes for Fe–S clusters results in lower amounts of holo-IscR and subsequently in less repression of IscR-dependent promoters (Fig. 1). Such a co-regulation was suggested for *Synechocystis* sp. PCC 6803: SufR is a DNA-binding regulator that coordinates an Fe–S cluster, autoregulates its own expression and expression of the *sufBCDS* operon and regulates reaction center biosynthesis (Yu et al. 2003; Wang et al. 2004; Shen et al. 2007).

Our results demonstrate that higher amounts of photosynthetic complexes in the wild type have only minor effects on the activity of the *isc-suf* promoters, including the IscR-dependent P2 and P3 promoters and rather resulted in decreased activity. Solely the activity of the weak P1 promoter was higher under anaerobic conditions. Activity of P2 that is strongly dependent on IscR (Remes et al. 2014; Nie et al. 2019) was not activated under conditions when production of photosynthetic complexes is highest. However, in mutants with reduced amounts of photosynthetic complexes P2 activity was elevated. Nevertheless, in all tested mutants activity of P2 was higher in presence of oxygen than under anaerobic conditions, when production of photosynthetic complexes is higher. This observation excludes a direct correlation of the amounts of photosynthetic complexes, the holo-IscR level and activity of the *isc-suf* promoters.

Only for the App11 mutant we observed, in addition to increased P2 activity, also increased activity of P1, P3, P4 and slightly increased activity for P5 compared to the wild type. While strains 2.4.1Δ*pucBApuc2BA* and BCHE lack components of the photosynthetic apparatus, strain App11 lacks the important redox- and light-dependent regulator AppA that affects all genes of the PpsR regulon including genes for the PpaA and PrrA regulators (Imam et al. 2015). PrrA targets a large regulon comprising photosynthesis genes but also genes not related to photosynthesis (Imam et al. 2014). Thus, other cellular changes than reduced amounts of photosynthetic complexes may cause the observed effect of App11 on *isc-suf* promoters.

The fact that formation of photosynthetic complexes does not result in marked changes in *isc-suf* promoter activities implies that post-transcriptional regulatory mechanisms lead to higher amounts of *isc-suf* mRNAs under low oxygen or anaerobic conditions, as previously shown (Remes et al. 2014). Indeed, our data demonstrate regulation at level of RNA stability. Elucidation of the mechanisms stabilizing *isc-suf* mRNA under low oxygen conditions is beyond the scope of this study. Many different enzymes, RNA binding proteins and stabilizing or destabilizing RNA elements determine the half-lives of mRNAs [e.g., (Evguenieva-Hackenberg and Klug 2011)]. In *R. sphaeroides* reduced RNase E activity strongly impacts phototrophic growth but does not affect doubling time under microaerobic conditions (Förstner et al. 2018), the reasons for this are not known to date. It was not possible to grow the *R. sphaeroides* strain with reduced RNase E activity under anaerobic dark conditions so that we could not test the contribution of RNase E to the prolonged *iscR* and *sufB* half-lives under these conditions.

In a mutant lacking the IscR regulator and showing reduced *iscS* mRNA levels and slightly increased mRNA levels for the *suf* genes (Remes et al. 2015), we observed impaired formation of photosynthetic complexes upon a drop of oxygen tension. This observation proofs some coupling between the Isc system and photosynthetic complex formation. Our results show that this effect is not due to altered levels of photosynthetic mRNAs. It is likely that impaired formation of Fe–S clusters by reduced expression of *suf* genes in the mutant limits the formation of photosynthetic complexes.

Another putative regulator linking Fe–S availability to formation of photosynthetic complexes in *R. sphaeroides* is the Fe–S protein FnrL. The activity of FnrL transcriptional regulators depends on the redox state and FnrL activates several genes related to photosynthesis under low oxygen tension (Zeilstra-Ryalls and Kaplan 1998). Since FnrL does not affect expression of *isc-suf* genes (Fig. S5), an influence on the production of Fe–S clusters is unlikely, but its role in Fe–S-dependent regulation of photosynthesis gene regulation deserves further analyses in the future.

In conclusion, the amounts of photosynthetic complexes and *isc-suf* operon expression are not coordinated at level of transcription in *R. sphaeroides*.

## Electronic supplementary material

Below is the link to the electronic supplementary material.

Supplementary file1 (DOCX 634 kb)

## References

[CR1] Arai H, Roh JH, Kaplan S (2008). Transcriptome dynamics during the transition from anaerobic photosynthesis to aerobic respiration in *Rhodobacter sphaeroides* 2.4.1. J Bacteriol.

[CR2] Braatsch S, Gomelsky M, Kuphal S, Klug G (2002). A single flavoprotein, AppA, integrates both redox and light signals in *Rhodobacter sphaeroides*. Mol Microbiol.

[CR3] Callister SJ, Nicora CD, Zeng X, Roh JH, Dominguez MA, Tavano CL, Monroe ME, Kaplan S, Donohue TJ, Smith RD, Lipton MS (2006). Comparison of aerobic and photosynthetic *Rhodobacter sphaeroides* 2.4.1 proteomes. J Microbiol Methods.

[CR4] Chory J, Donohue TJ, Varga AR, Staehelin LA, Kaplan S (1984). Induction of the photosynthetic membranes of *Rhodopseudomonas sphaeroides*: biochemical and morphological studies. J Bacteriol.

[CR5] Clayton RK (1966). The bacterial photosynthetic reaction center. Brookhaven Symp Biol.

[CR6] Crofts AR, Meinhardt SW, Jones KR, Snozzi M (1983). The role of the quinone pool in the cyclic electron-transfer chain of *Rhodopseudomonas Sphaeroides*: a modified Q-cycle mechanism. Biochim Biophys Acta.

[CR7] Evguenieva-Hackenberg E, Klug G (2011). New aspects of RNA processing in prokaryotes. Curr Opin Microbiol.

[CR8] Förstner KU, Reuscher CM, Haberzettl K, Weber L, Klug G (2018). RNase E cleavage shapes the transcriptome of *Rhodobacter sphaeroides* and strongly impacts phototrophic growth. Life Sci Alliance.

[CR9] Georg J, Kostova G, Vuorijoki L, Schön V, Kadowaki T, Huokko T, Baumgartner D, Müller M, Klähn S, Allahverdiyeva Y, Hihara Y, Futschik ME, Aro EM, Hess WR (2017). Acclimation of oxygenic photosynthesis to iron starvation is controlled by the sRNA IsaR1. Curr Biol.

[CR10] Giel JL, Rodionov D, Liu MZ, Blattner FR, Kiley PJ (2006). IscR-dependent gene expression links iron-sulphur cluster assembly to the control of O_2_-regulated genes in *Escherichia coli*. Mol Microbiol.

[CR11] Gomelsky M, Kaplan S (1997). Molecular genetic analysis suggesting interactions between AppA and PpsR in regulation of photosynthesis gene expression in *Rhodobacter sphaeroides 2.4.1*. J Bacteriol.

[CR12] Han Y, Meyer MH, Keusgen M, Klug G (2007). A haem cofactor is required for redox and light signalling by the AppA protein of *Rhodobacter sphaeroides*. Mol Microbiol.

[CR13] Imam S, Noguera DR, Donohue TJ (2014). Global analysis of photosynthesis transcriptional regulatory networks. PLoS Genet.

[CR14] Imam S, Noguera DR, Donohue TJ (2015). An integrated approach to reconstructing genome-scale transcriptional regulatory networks. PLoS Comput Biol.

[CR15] Johnson DC, Dean DR, Smith AD, Johnson MK (2005). Structure, function, and formation of biological iron-sulfur clusters. Annu Rev Biochem.

[CR16] Kessler D, Papenbrock J (2005). Iron-sulfur cluster biosynthesis in photosynthetic organisms. Photosynth Res.

[CR17] Kovach ME, Elzer PH, Hill DS, Robertson GT, Farris MA, Roop RM, Peterson KM (1995). Four new derivatives of the broad-host-range cloning vector pBBR1MCS, carrying different antibiotic-resistance cassettes. Gene.

[CR18] Lee KC, Yeo WS, Roe JH (2008). Oxidant-responsive induction of the *suf* operon, encoding a Fe-S assembly system, through Fur and IscR in *Escherichia coli*. J Bacteriol.

[CR19] Masuda S, Bauer CE (2002). AppA is a blue light photoreceptor that antirepresses photosynthesis gene expression in *Rhodobacter sphaeroides*. Cell.

[CR20] Nie X, Remes B, Klug G (2019). Multiple Sense and Antisense Promoters Contribute to the Regulated Expression of the *isc-suf* Operon for Iron-Sulfur Cluster Assembly in *Rhodobacter*. Microorganisms.

[CR21] Peuser V, Remes B, Klug G (2012). Role of the Irr Protein in the Regulation of Iron Metabolism in *Rhodobacter sphaeroides*. PLoS ONE.

[CR22] Pfaffl MW (2001). A new mathematical model for relative quantification in real-time RT-PCR. Nucleic Acids Res.

[CR23] Purvis DJ, Theiler R, Niederman RA (1990). Chromatographic and protein chemical analysis of the ubiquinol-cytochrome c2 oxidoreductase isolated from *Rhodobacter sphaeroides*. J Biol Chem.

[CR24] Py B, Barras F (2010). Building Fe-S proteins: bacterial strategies. Nat Rev Microbiol.

[CR25] Remes B, Berghoff BA, Förstner KU, Klug G (2014). Role of oxygen and the OxyR protein in the response to iron limitation in *Rhodobacter sphaeroides*. BMC Genomics.

[CR26] Remes B, Eisenhardt BD, Srinivasan V, Klug G (2015). IscR of *Rhodobacter sphaeroides* functions as repressor of genes for iron-sulfur metabolism and represents a new type of iron-sulfur-binding protein. Microbiologyopen.

[CR27] Selvi MT, Sharma R (2008). Cell maturity gradient determines light regulated accumulation of proteins in pearl millet leaves. Physiol Mol Biol Plants.

[CR28] Sener MK, Olsen JD, Hunter CN, Schulten K (2007). Atomic-level structural and functional model of a bacterial photosynthetic membrane vesicle. Proc Natl Acad Sci USA.

[CR29] Shen G, Balasubramanian R, Wang T, Wu Y, Hoffart LM, Krebs C, Bryant DA, Golbeck JH (2007). SufR coordinates two [4Fe-4S]^2+, 1+^ clusters and functions as a transcriptional repressor of the *sufBCDS* operon and an autoregulator of *sufR* in cyanobacteria. J Biol Chem.

[CR30] Sirijovski N, Mamedov F, Olsson U, Styring S, Hansson M (2007). *Rhodobacter capsulatus* magnesium chelatase subunit BchH contains an oxygen sensitive iron-sulfur cluster. Arch Microbiol.

[CR31] Trumpower BL (1990). Cytochrome bc1 complexes of microorganisms. Microbiol Rev.

[CR32] Wang T, Shen G, Balasubramanian R, McIntosh L, Bryant DA, Golbeck JH (2004). The sufR gene (*sll0088* in *Synechocystis sp*. strain PCC 6803) functions as a repressor of the *sufBCDS* operon in iron-sulfur cluster biogenesis in cyanobacteria. J Bacteriol.

[CR33] Yu J, Shen G, Wang T, Bryant DA, Golbeck JH, McIntosh L (2003). Suppressor mutations in the study of photosystem I biogenesis: *sll0088* is a previously unidentified gene involved in reaction center accumulation in *Synechocystis sp.* strain PCC 6803. J Bacteriol.

[CR34] Zang SS, Jiang HB, Song WY, Chen M, Qiu BS (2017) Characterization of the sulfur-formation (*suf*) genes in *Synechocystis sp*. *PCC 6803* under photoautotrophic and heterotrophic growth conditions. Planta 246(5):927–938. 10.1007/s00425-017-2738-0 10.1007/s00425-017-2738-0 28710587

[CR35] Zeilstra-Ryalls JH, Kaplan S (1998). Role of the *fnrL* gene in photosystem gene expression and photosynthetic growth of *Rhodobacter sphaeroides* 2.4.1. J Bacteriol.

[CR36] Zeller T, Klug G (2004). Detoxification of hydrogen peroxide and expression of catalase genes in *Rhodobacter*. Microbiology.

[CR37] Zeng X, Roh JH, Callister SJ, Tavano CL, Donohue TJ, Lipton MS, Kaplan S (2007). Proteomic characterization of the *Rhodobacter sphaeroides* 2.4.1 photosynthetic membrane: identification of new proteins. J Bacteriol.

