# Supplementary material for: Interplay between formation of photosynthetic complexes and expression of genes for iron–sulfur cluster assembly in Rhodobacter sphaeroides?
Source: Photosynth Res. 2020 Oct 16;147(1):39–48. doi: 10.1007/s11120-020-00789-w (PMC7728643; doi:10.1007/s11120-020-00789-w)
Supplement: Supplementary file 1 — Supplementary file1 (DOCX 634 kb) [file 11120_2020_789_MOESM1_ESM.docx]

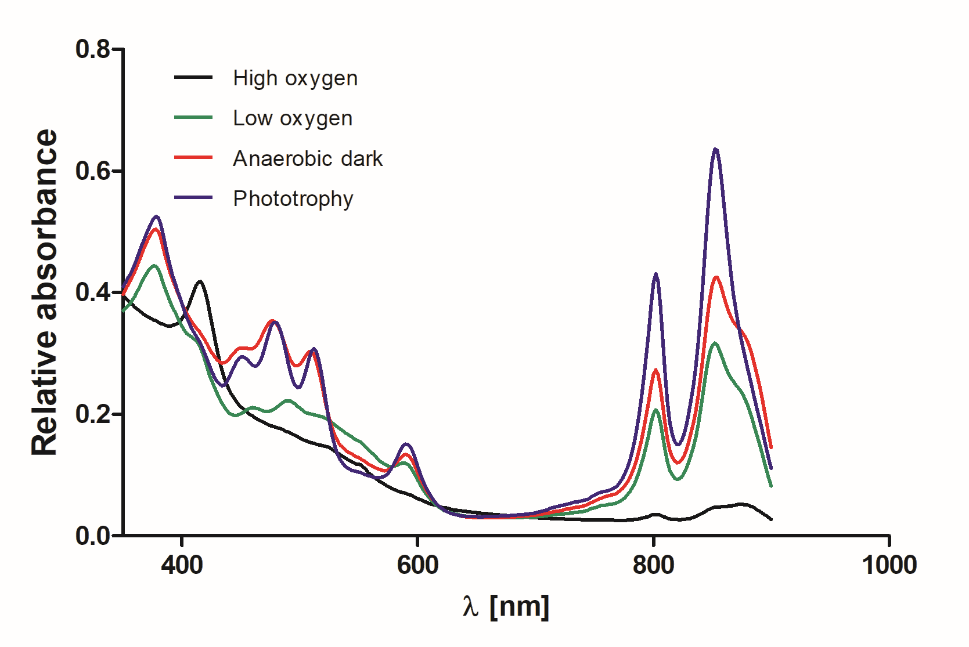


**Fig. S1** Whole cell spectra of the *R. sphaeroides* wild type 2.4.1 grown at the indicated conditions. The same amounts of cells were applied for each condition.

**
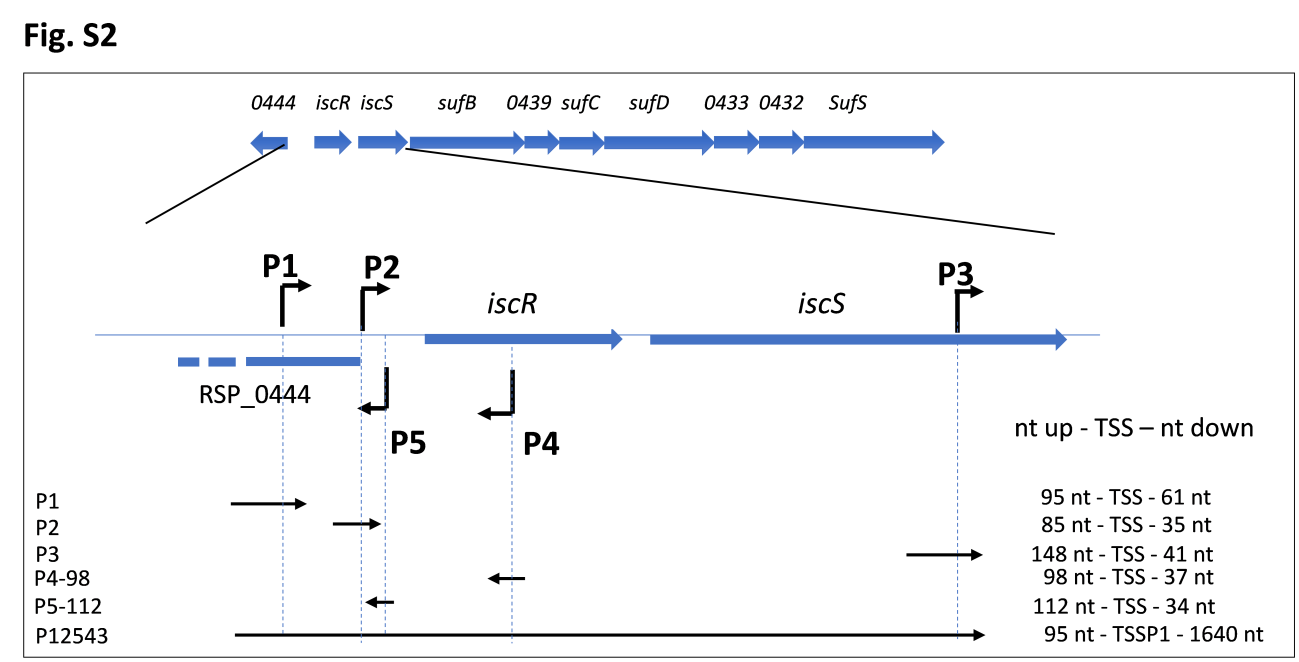
**

**Fig. 2** Scheme of the *isc-suf* operon of *R. sphaeroides*. The positions of 5 verified promotors ared indicated. The horizontal arrows represent the DNA fragments transcriptionally fused to the *lac*Z gene at the position of the arrow head. TSS: transcriptional start site as determined by differential RNAseq in ([Remes et al. 2017](#_ENREF_4)).


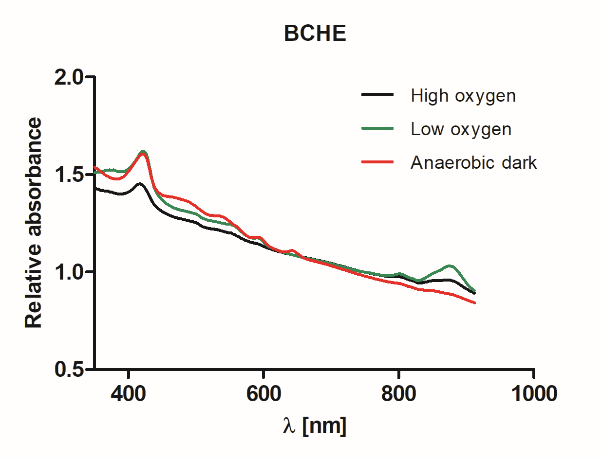

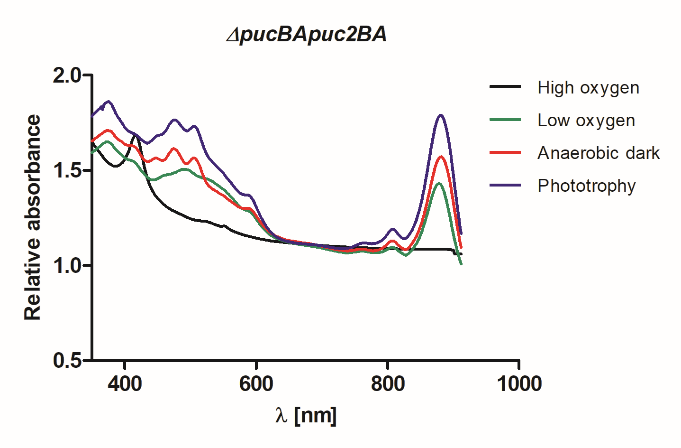

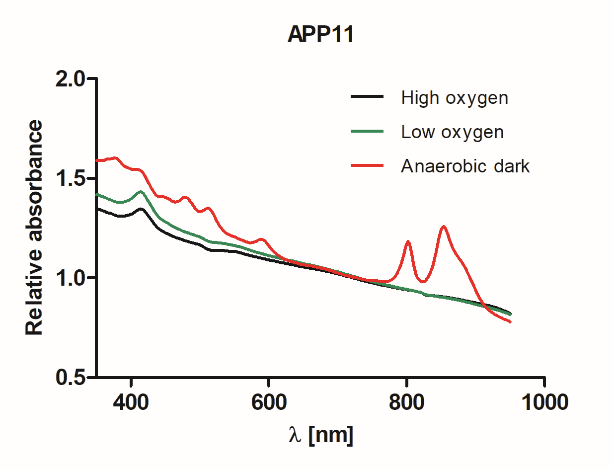


**Fig. S3** Whole cell spectra of various mutant strains under different growth conditions. The identical amounts of cells were applied for the different strains and conditions.


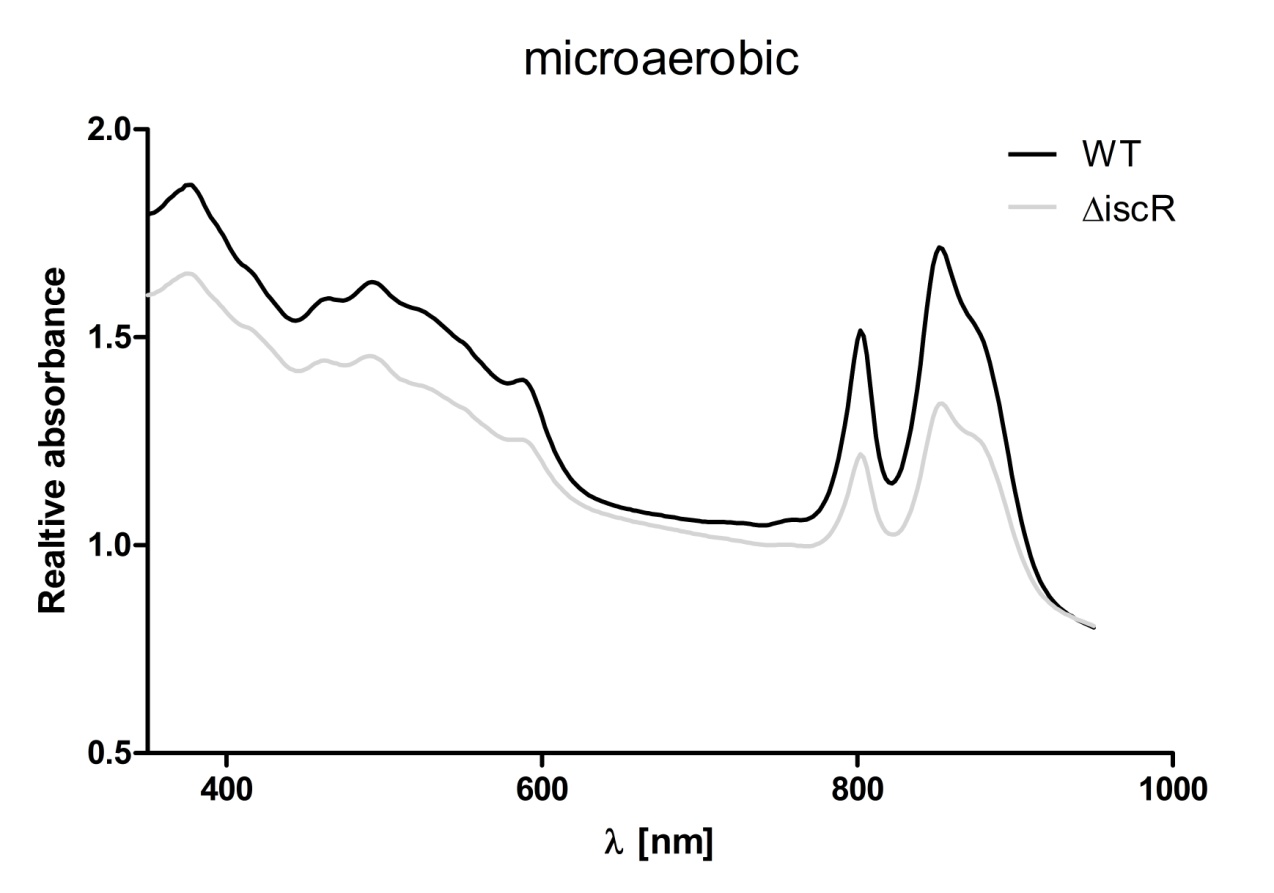


**Fig. S4** Whole cell spectra of wild type and *iscR* mutant under low oxygen. The identical amounts of cells were applied for the two strains.


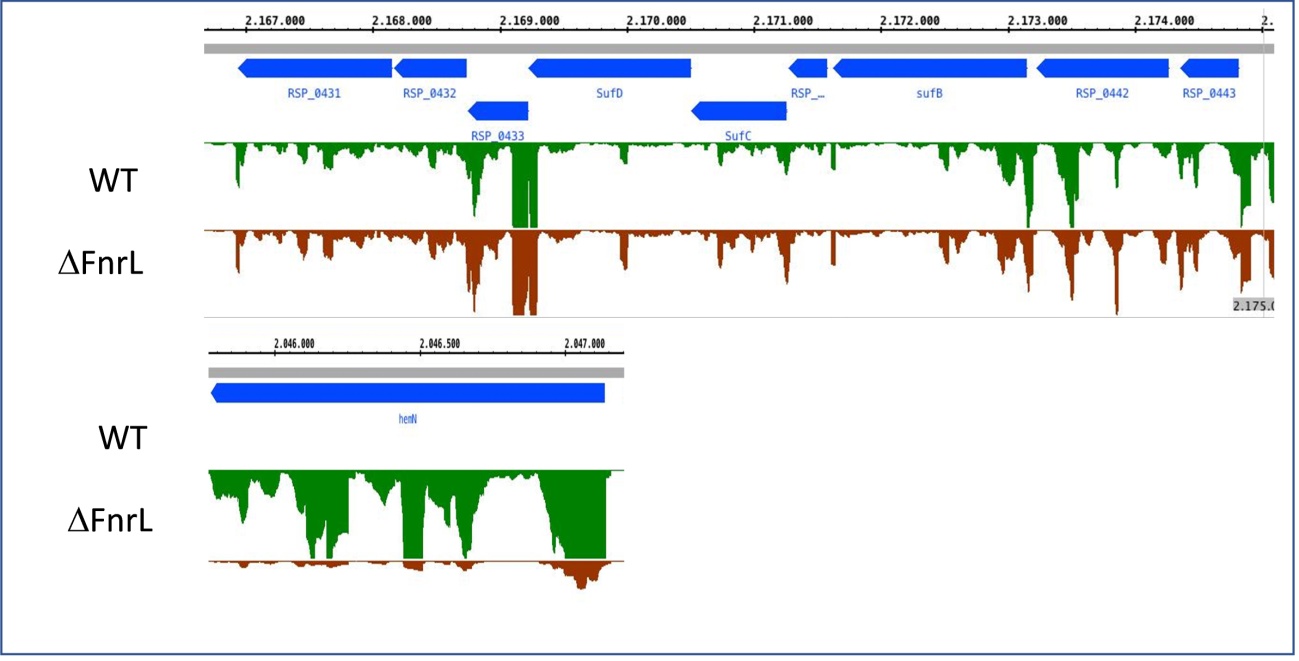


**Fig. S5** Screen shots from the Integrated Genome Browser showing RNAseq reads for the *isc-suf* operon (upper panel) and the *hemN* gene (lower panel). Cultures were grown in iron replete medium under low oxygen conditions and exponential phase cells were used for RNA isolation. Identical scales (0-100 reads) were applied to all samples. DESeq analyses revealed 18-fold higher normalized read number for hemN in the wild type compared to the mutant. The individual *isc-suf* genes had 0.9 to 1.5-fold read numbers in the wild type compared to the mutant with an overall average of 1.3-fold. There is also no influence of FnrL on *isc-suf* genes under iron repletion (not shown).

**Table S1-** *R. sphaeroides* and *E. coli* strains/plasmids used in this study

| **Strains/Plasmid names** | **Relevant features** | **References** |
| --- | --- | --- |
| *R. sphaeroides* |  |  |
| 2.4.1 | *Rhodobacter sphaeroides* wild type | (van Niel, 1944) |
| *∆pucBA2BA* | *puc1BA* and *puc2BA* deletion strain | ([Zeng et al. 2003](#_ENREF_7)) |
| BCHE | Km^r^, *bchE* deletion strain | ([Gomelsky and Kaplan 1995](#_ENREF_1)) |
| APP11 | Tp^r^, *appA* deletion strain | ([Gomelsky and Kaplan 1995](#_ENREF_1)) |
| *E. coli* |  |  |
| JM109 | Host strain for cloning procedures | ([Yanisch-Perron et al. 1985](#_ENREF_6)) |
| S17-1 | Strain for diparental conjugation, tra^+^ | ([Simon et al. 1986](#_ENREF_5)) |
| pBBR1-MCS3-*lacZ* | Tc^r^, Broad-host-range cloning vector | ([Kovach et al. 1995](#_ENREF_2)) |
| pBBR1-MCS3-*lacZ*-P1 | Tc^r^, Promoter 1 (95bp) from *isc suf* operon on pBBR1-MCS3-*lacZ* | This study |
| pBBR1-MCS3-*lacZ*-P2 | Tc^r^, Promoter 2 from *isc suf* operon on pBBR1-MCS3-*lacZ* | ([Nie et al. 2019](#_ENREF_3)) |
| pBBR1-MCS3-*lacZ*-P3 | Tc^r^, Promoter 3 from *isc suf* operon on pBBR1-MCS3-*lacZ* | ([Nie et al. 2019](#_ENREF_3)) |
| pBBR1-MCS3-*lacZ*-P4-98 | Tc^r^, Promoter 4(98 nt upstream) from *isc suf* operon on pBBR1-MCS3-*lacZ* | ([Nie et al. 2019](#_ENREF_3)) |
| pBBR1-MCS3-*lacZ*-P5-112 | Tc^r^, Promoter 5(112 nt upstream) from *isc suf* operon on pBBR1-MCS3-*lacZ* | ([Nie et al. 2019](#_ENREF_3)) |
| pBBR1-MCS3-*lacZ*-P12543 | Tc^r^, Promoter 1, 2, 5, 4 and 3 (1736bp) from *isc suf* operon on pBBR1-MCS3-*lacZ* | This study |

Sp^r^, spectinomycin-resistant; Tp^r^, trimethoprim-resistant; Km^r^, kanamycin resistant; Tc^r^, tetracycline-resistant; when required, antibiotics were added in the following concentrations: tetracycline-resistant (2 μg·ml^-1^), kanamycin (25 μg·ml^-1^) and trimethoprim (50 μg·ml^-1^) for *R. sphaeroides.*

**Table S2** – Oligonucleotides used in this study

| Name | Sequence | Purpose |
| --- | --- | --- |
| P1_fwd | ACTAtctaGATGCCCTGATCGTACTCGC | forward primer for promoter 1-95 of *isc-suf*-operon cloning |
| P1_rev | ACTACTGCAGTTGCCATCGTGCTGCAC | reverse primer for promoter 1 of *isc-suf*-operon cloning |
| P2_fwd | ACTATCTAGAAAATCACTTCGGGCATCGC | forward primer for promoter 2 of *isc-suf*-operon cloning |
| P2_rev | ACTACTGCAGTCGTTACGGTTCCGGGC | reverse primer for promoter 2 of *isc-suf*-operon cloning |
| P3_fwd | ACTACCCGGGAAAACCTGATCGGGATCGC | forward primer for promoter 3 of *isc-suf*-operon cloning |
| P3_rev | ACTACTGCAGTGACGAGGCAGAGCGTGTT | reverse primer for promoter 3 of *isc-suf*-operon cloning |
| P4as_fwd | ACTATCTAGACAAGGATTGTGCACGCGAG | forward primer for promoter 4(98 nt upstream) of *isc-suf*-operon cloning |
| P4as_rev | ACTACTGCAGCGGCTACAAGCTCGCGC | reverse primer for promoter 4 of *isc-suf*-operon cloning |
| P5as_fwd | ACTATCTAGAGCGCGAGATCCACCAGC | forward primer for promoter 5(112 nt upstream) of *isc-suf*-operon cloning |
| P5as_rev | ACTACTGCAGAACCATTCCACCTGGGCG | reverse primer for promoter 5 of *isc-suf*-operon cloning |
| pucB_fwd | CGAAGCCGAAGAAGTTCA | RSP_0314 (*pucB*) real-time RT-PCR |
| pucB_rev | TTCACCACGAGCCAGATT | RSP_0314 (*pucB*) real-time RT-PCR |
| pufL_fwd | ACACCTACGGCAACTTCC | RSP_0257 (*pufL*) real-time RT-PCR |
| pufL _rev | ATCGAGTAGCCGACCAGA | RSP_0257 (*pufL*) real-time RT-PCR |
| puhA_fwd | AAGCCCAAGACCTTCATCCT | RSP_0291 (*puhA*) real-time RT-PCR |
| puhA _rev | TCATCGGCTTGATCTTGTTG | RSP_0291 (*puhA*) real-time RT-PCR |
| bchY_fwd | CAATTCGGAATCGCTCGT | RSP_0261 (*bchY*) real-time RT-PCR |
| bchY _rev | ACGCAGAGGTTCGTCACC | RSP_0261 (*bchY*) real-time RT-PCR |
| crtI_fwd | CGAGATCCTCGTCGAGAA | RSP_0271 (*crtI*) real-time RT-PCR |
| crtI _rev | CAGAAGCCGCATGTAGGT | RSP_0271 (*crtI*) real-time RT-PCR |
| hemA_fwd | GCACCACGCTCTATCACA | RSP_2984 (*hemA*) real-time RT-PCR |
| hemA _rev | CAGGTCGTCGAGGTCATT | RSP_2984 (*hemA*) real-time RT-PCR |
| hemP_fwd | ATGACCGACCCGATGGAG | RSP_6006 (*hemP*) real-time RT-PCR |
| hemP _rev | AGTAGATCTGCCCGTCGAG | RSP_6006 (*hemP*) real-time RT-PCR |
| RT_RSP_0799_A | GAACAATTACGCCTTCTC | RSP_0799 (*gloB*) real-time RT-PCR |
| RT_RSP_0799_B | CATCAGCTGGTAGCTCTC | RSP_0799 (*gloB*) real-time RT-PCR |
| RT_RSP_1669_A  RT_RSP_1669_B | ATCGCGGAAGAGACCCAGAG  GAGCAGCGCCATCTGATCCT | RSP_1669 (*rpoZ*) real-time RT-PCR  RSP_1669 (*rpoZ*) real-time RT-PCR |
| RT_RSP_0440_A | ACATCGTGCGGCTGATCT | RSP_0440 (*sufB*) real-time RT-PCR |
| RT_RSP_0440_B | CGGCTTCACATCCATGCT | RSP_0440 (*sufB*) real-time RT-PCR |
| RT_RSP_0443_A  RT_RSP_0443_B | GGTGGAAGAGACGCTCAA  ATAGACATGCGCCGAGAC | RSP_0443 (*iscR*) real-time RT-PCR  RSP_0443 (*iscR*) real-time RT-PCR |

Gomelsky M and Kaplan S (1995) *appA*, a novel gene encoding a trans-acting factor involved in the regulation of photosynthesis gene expression in *Rhodobacter sphaeroides* 2.4.1. J Bacteriol 177(16): 4609-4618. doi 10.1128/jb.177.16.4609-4618.1995

Kovach ME, Elzer PH, Hill DS, Robertson GT, Farris MA, Roop RM, 2nd and Peterson KM (1995) Four new derivatives of the broad-host-range cloning vector pBBR1MCS, carrying different antibiotic-resistance cassettes. Gene 166(1): 175-176. doi 10.1016/0378-1119(95)00584-1

Nie X, Remes B and Klug G (2019) Multiple Sense and Antisense Promoters Contribute to the Regulated Expression of the *isc-suf* Operon for Iron-Sulfur Cluster Assembly in *Rhodobacter*. Microorganisms 7(12). doi 10.3390/microorganisms7120671

Remes B, Rische-Grahl T, Müller KMH, Förstner KU, Yu SH, Weber L, Jäger A, Peuser V and Klug G (2017) An RpoHI-Dependent Response Promotes Outgrowth after Extended Stationary Phase in the Alphaproteobacterium *Rhodobacter sphaeroides*. J Bacteriol 199(14). doi 10.1128/JB.00249-17

Simon R, Oconnell M, Labes M and Pühler A (1986) Plasmid Vectors for the Genetic-Analysis and Manipulation of *Rhizobia* and Other Gram-Negative Bacteria. Method Enzymol 118: 640-659 doi 10.1016/0076-6879(86)18106-7

van Niel CB (1944) The culture, general physiology, morphology, and classification of the non-sulfur purple and brown bacteria. Bacteriological reviews 8: 118

Yanisch-Perron C, Vieira J and Messing J (1985) Improved M13 phage cloning vectors and host strains: nucleotide sequences of the M13mp18 and pUC19 vectors. Gene 33(1): 103-119. doi 10.1016/0378-1119(85)90120-9

Zeng X, Choudhary M and Kaplan S (2003) A second and unusual *pucBA* operon of *Rhodobacter sphaeroides* 2.4.1: genetics and function of the encoded polypeptides. J Bacteriol 185(20): 6171-6184. doi 10.1128/jb.185.20.6171-6184.2003
